# Reframing vaccine narrative: a co-production study of a media campaign intervention to address childhood vaccine hesitancy among Nigerian parents and caregivers

**DOI:** 10.3389/fpubh.2026.1857030

**Published:** 2026-07-02

**Authors:** Tarela Juliet Ike, Dung Ezekiel Jidong, Callistar Kidochukwu Obi, Maureen Iru Ntaji, Mieyebi Lawrence Ike, Evangelyn Ebi Ayobi, Peremi Richmond Ike, Rukevwe Francis Doghor, Christopher Francis, Kelvin Odeyovwi Ayobi, Nusrat Husain

**Affiliations:** 1 School of Social Sciences, Humanities and Law, Teesside University, Middlesbrough, United Kingdom; 2 Division of Psychology and Mental Health, University of Manchester, Manchester, United Kingdom; 3 Department of Economics, Faculty of the Social Sciences, Delta State University, Abraka, Nigeria; 4 Department of Community Medicine, Delta State University Teaching Hospital, Oghara, Delta, Nigeria; 5 Western Governors University, Millcreek, UT, United States; 6 Manchester Global Foundation, London, United Kingdom; 7 Tare Wyd Legal Chambers, Warri, Nigeria; 8 Beryl Foundation, London, United Kingdom; 9 Nasarawa State University, Keffi, Nigeria; 10 Mersey Care NHS Foundation Trust, Liverpool, United Kingdom

**Keywords:** caregiver, childhood vaccine hesitancy, Nigeria, parents, vaccine

## Abstract

**Introduction:**

Childhood vaccine hesitancy is a public health concern. Nigeria is one of the countries with the highest rates of zero-dose childhood vaccination. This study makes an original contribution by adopting a co-production approach underpinned by interpretative phenomenological analysis (IPA) to meaningfully inform the co-production of a media campaign intervention with Nigerian parents/ caregivers whose child(ren) are not, or only partially, up to date with routine immunizations aimed at reducing childhood vaccine hesitancy and promoting uptake.

**Methods:**

A total of 10 parents or caregivers whose children were not up to date with their vaccinations were recruited for the study and participated in a focus group discussion. Data were analyzed using IPA.

**Results:**

The findings reveal that hostility, misinformation, and the breakdown of trust in vaccines, alongside faith, tradition, and the lived logic of alternative protection, intersect to exacerbate hesitancy. The findings also reveal that clarity, reassurance, and empowerment in vaccine communication, underpinned by gendered voices, can build trust in vaccine messaging and encourage uptake.

**Discussion:**

The study offers important insights for policymakers and public health communication strategies, underscoring the need for culturally appropriate media campaign interventions that address vaccine-related concerns and foster uptake.

## Introduction

Childhood vaccine hesitancy remains a significant global public health challenge, with millions of children missing routine immunizations each year. In 2023 alone, 14.5 million children worldwide received no routine childhood vaccines, a rise from the previous year, reflecting persistent mistrust, misinformation, and access barriers in many regions (1). Nigeria represents one of the most affected regions, with over 2.1 million zero-dose children, the largest population of unvaccinated children in Africa (2). This high burden is often driven by factors such as misinformation, poor access to health services, and sociocultural beliefs. Studies highlight that despite ongoing efforts, Nigeria continues to struggle with low routine immunization coverage and a growing number of under-immunized children (3). Non-vaccination can lead to adverse effects, including increased outbreaks of vaccine-preventable diseases (4), higher child morbidity and mortality (5), and setbacks in achieving national and global health targets (6). As unvaccinated populations grow, herd immunity weakens, allowing diseases such as measles, polio, and diphtheria to resurge, placing the most vulnerable children at a greater risk. For example, the poliovirus seroprevalence meta-analysis provides a useful benchmark for why population-level immunity gaps matter for policy and why communication interventions should be linked to measurable coverage and protection goals (7). This is further amplified following the COVID-19 pandemic and the urgent need for post-COVID service adaptation, including public health communication and service delivery after major health-system shocks (8). However, a gap remains in the collaborative development of a media campaign intervention that uses an interpretative phenomenological analytical approach, with parents/caregivers of children who are unvaccinated or not up to date with their immunizations in order to aid vaccine uptake and prevent hesitancy.

A growing body of literature stresses that poor childhood vaccination uptake in Nigeria reflects a persistent, deeply rooted public health challenge shaped by structural (9), social (10), and geographic factors (11). Similar challenges are evident in other countries as well. For example, in Italy, vaccine uptake is also shaped by subgroup differences, risk perception, and system-level monitoring, even beyond the context of childhood immunizations (8). In Nigeria, the extent of the problem is further delineated by the Nigeria Demographic and Health Survey (12), which reports that 31% of children aged 12–23 months received no routine vaccines, leaving them vulnerable to preventable diseases and undermining national immunization goals. Routine vaccines such as BCG, polio (OPV/IPV), DPT-HepB-Hib, pneumococcal conjugate vaccine, rotavirus, yellow fever, meningitis, and measles remain unevenly accessed across the country (13). This gap contributes to recurrent outbreaks and high child morbidity and mortality, reinforcing the urgency of understanding and addressing the factors behind the low vaccination uptake.

In addition, socio-economic constraints reduce caregivers’ ability to attend immunization sessions. These include poverty (14), transportation costs (15), and competing livelihood demands. Cultural informational barriers also contribute to hesitancy or non-attendance. Such barriers include low maternal education, misinformation (16), and limited awareness of vaccine schedules (17). Nigeria is a country entrenched in cultural norms (18–20). These factors collectively interact to create chronic pockets of under-immunization that persist across decades. However, there remains a gap in co-producing interventions to address this significant issue.

In light of the preceding gaps, this study makes an original and significant contribution through an IPA-informed co-production approach. IPA offers a significant, deeply human-centered foundation for co-producing media campaign interventions with Nigerian parents whose children are not, or only partially, up to date with routine vaccinations. IPA, rooted in lived experience, offers a unique lens for addressing the complex challenges of childhood vaccine hesitancy. By focusing on the lived experiences of parents and caregivers, IPA uncovers the personal meanings, emotions, cultural influences, and social pressures that shape vaccine hesitancy—insights often overlooked by quantitative approaches. This depth matters in Nigeria, where hesitancy is frequently tied to misinformation, mistrust, religious narratives, and prior negative encounters with the health system.

Furthermore, IPA enables a move beyond surface-level assumptions and instead supports understanding how parents interpret vaccination messages, why they hesitate, and what forms of communication feel respectful, trustworthy, and empowering to them. These insights become the backbone of co-production: parents are not passive recipients but active partners whose perspectives guide message framing, tone, imagery, and delivery channels. In a context where many caregivers rely on social networks and community voices for health information, IPA-informed co-production ensures that media campaigns resonate authentically, address real fears, and reflect the linguistic, cultural, and emotional realities of families. Ultimately, this approach strengthens the credibility and relevance of vaccine communication, helping to rebuild trust and reduce hesitancy by creating interventions that parents recognize as their own. Hence, the research question.

- How can parents or caregivers whose child(ren) are not up to date, or only or partially up to date, with immunizations inform the development and co-production of a media campaign intervention that effectively promotes childhood vaccine uptake and reduces hesitancy?

For this study, we define vaccine hesitancy as the unwillingness to receive a vaccine despite its availability. To address the research question, the next section highlights the methodology adopted. This is followed by the findings and a discussion linked to the literature.

## Methodology

### Study design and theoretical framework

This study was underpinned by a qualitative approach and a case study design. It was also informed by an interpretivist epistemology, which emphasizes the subjectivity, complexity, and dynamism of social reality (21), alongside a constructivist ontology that emphasizes the social construction of knowledge (22). Interpretivist epistemology and constructionist ontology provide a coherent lens for studying how media campaigns can be co-produced with Nigerian parents experiencing childhood vaccine hesitancy. An interpretivist stance assumes that parents’ beliefs, fears, and motivations around vaccination are shaped by subjective meanings rather than fixed, measurable variables. This makes their lived experiences, cultural narratives, and interpersonal influences central to understanding why hesitancy persists and how communication can meaningfully address it. A constructionist ontology complements this by viewing vaccine hesitancy not as an inherent trait but as socially produced through interactions among caregivers, communities, health workers, religious leaders, and media environments. Together, these perspectives position the co-production of a media intervention as a process of jointly creating meaning—where parents are not passive recipients of health messages but active contributors whose interpretations shape the campaign’s design, tone, and relevance.

### Ethics statement

In line with global best practice and prior to the study commencement, ethical approval was sought from and granted by the Jos University Teaching Hospital Research Ethics Committee, with ethics approval number JUTH/DCS/IREC/127/XXXI/3322. The study was conducted in accordance with the Helsinki Declaration. All participants were provided with full information on the study’s aims, objectives, and methods prior to taking part. Participants were also informed of how their data will be used, including confidentiality, grounds for a confidentiality breach, anonymity, and their right to withdraw without being obliged to provide a reason. All participants voluntarily consented and completed the electronic consent form prior to participation.

### Participant demographics and sampling

A purposive sampling strategy was employed to recruit participants who met the inclusion criteria regarding childhood vaccine hesitancy. Recruitment drew on established relationships with third-sector organizations, including non-governmental and faith-based groups such as churches and mosques, as well as local community networks, enabling access to caregivers actively engaged in child-health decision-making. To further expand the sample and reach individuals with similar lived experiences, participants were also invited to recommend others who met the study criteria, supporting broader and more diverse participation.

In total, 10 parents/caregivers with lived experience of vaccine hesitancy were recruited for this study. Of the 10, 4 were men and 6 were women. Five had attained a secondary level of education, while the other five indicated a tertiary level of education. Regarding employment status, six were employed, while the other four were unemployed. The participants were recruited from Delta State, Nigeria, and represented states such as Delta, Adamawa, Osun, Ogun, Plateau, and Lagos. The participants were between 18 and 60 years old.

The rationale for the sample size of 10 participants is that it is methodologically appropriate for IPA, which prioritizes depth of idiographic engagement over numerical breadth. Foundational IPA scholarship consistently recommends small, purposive samples—typically 6–12 participants—to enable detailed experiential analysis (30, 31). Our dataset met this threshold and provided sufficient experiential richness, evidenced by theoretical sufficiency at the level of meaning-making rather than surface-level thematic repetition. As IPA emphasizes “theoretical sufficiency” rather than statistical saturation (32), we judged the dataset to be adequately rich once no new interpretative layers emerged during iterative coding and reflexive memoing.

Qualitative research does not claim population representativeness but supports transferability through the provision of thick description (33). To enhance transferability, our diverse sample—spanning geography and education—captures varied parental meaning-making, reflecting the broader issues surrounding childhood vaccine hesitancy, including zero-dose children and poor uptake documented by Mohammed et al. (2), thereby demonstrating how our findings may be relevant beyond the study participants.

### Data collection

To ensure robust data collection, this study first convened a steering committee comprising parents and caregivers with lived experience of either delaying or not completing childhood vaccinations. This group played a central role in shaping the initial focus group questions. An early phase of stakeholder and community engagement further informed this process, during which participants highlighted the importance of designing a media-based intervention and offered insights into the types of questions needed to ensure the campaign genuinely reflected the needs of the affected population. The collective contributions from these engagements guided the development of the focus group guide, which was subsequently submitted for ethical review and approved by the Jos University Teaching Hospital Health Research Ethics Committee.

Two 90-min focus groups, each comprising five participants, were conducted for this study. The rationale for adopting focus groups as a means of data collection was that they enable the rich, collaborative exploration of lived experiences that IPA seeks to understand (23), offering a depth of experiential data that is well suited to examining how parents make sense of being not up to date with childhood vaccinations. This approach aligns with IPA’s strengths in capturing personal meaning-making within a shared context, particularly when adapted for group discussions to elicit nuanced accounts of complex health-related issues. Examples of questions in the focus group guide include the following: What are your experiences with childhood vaccine hesitancy? What are your views on the role of the media in changing or shaping attitudes toward childhood vaccine hesitancy? What media platforms do you think parents or caregivers are more likely to use and engage with? What factors do you think we need to consider when designing the media intervention to increase its likelihood of improving parents’ and caregivers’ childhood vaccination uptake? Which key stakeholders (e.g., pastors, imams, etc.) do you think need to be involved in making the media campaign a success? What do you think should be the content of the media campaign intervention? Who do you think should deliver the media message in the media campaign, and why? Are there any thoughts you would like to add that may not have been covered in this focus group?

The focus group sessions were facilitated by a female participant, an experienced MSc health research fellow with lived experience, who had been trained by the research team in focus group interviewing techniques. Facilitation was further supported by a senior colleague with doctoral-level expertise and extensive experience working with the target population, alongside additional senior members of the project team, including a psychologist.

### Data analysis

The IPA was used to explore how parents in Nigeria make sense of childhood vaccine hesitancy and how they experience the co-production of a media campaign designed to address it. IPA is grounded in the view that meaning is constructed through lived experience, making it well-suited to examine the emotional, cultural, and relational dynamics that shape vaccine decision-making. The analysis followed the core IPA stages: first, each transcript was read repeatedly to establish familiarity and to identify initial descriptive, linguistic, and conceptual notes. These notes were then developed into emergent themes that captured each participant’s meaning-making. Themes were clustered into higher-order categories through an iterative process that alternated between individual accounts and the developing analytic structure. This idiographic approach ensured that each parent’s perspective was examined in depth before patterns were identified across the dataset.

Consistent with IPA’s interpretivist foundations, the analysis acknowledged the double hermeneutic: participants interpret their own experiences, and the researcher interprets those interpretations. Reflexive memos were maintained throughout the analytic process to document assumptions, emotional reactions, and positional influences—particularly important given the cultural and public health sensitivities surrounding vaccine hesitancy in Nigeria. These memos supported transparency and helped ensure that interpretations remained grounded in participants’ accounts rather than the researchers’ expectations.

To enhance analytic rigor while respecting IPA’s interpretive depth, coding credibility was strengthened through a collaborative coding process rather than statistical agreement metrics that would conflict with IPA’s idiographic ethos. A second trained qualitative researcher independently coded a purposive subset of transcripts (typically 20–25%) using the same IPA framework. The two coders then met to compare interpretations, discuss points of divergence, and refine theme boundaries. Rather than seeking numerical concordance, the goal was to strengthen conceptual clarity, challenge potential blind spots, and ensure that themes were credible, defensible, and grounded in the data. Discrepancies were resolved through dialog, with final decisions guided by the richness and coherence of participants’ narratives. This approach balanced IPA’s emphasis on interpretive nuance with the need for methodological transparency and trustworthiness. Following these discussions, the full dataset was revisited to test the refined thematic structure against all transcripts. Themes were adjusted, merged, or expanded as appropriate to ensure they captured both shared patterns and important divergences in parents’ experiences. The final thematic framework was supported by verbatim extracts selected to illustrate the depth, complexity, and emotional texture of participants’ meaning-making. This iterative, interpretive, and collaborative process produced an analysis that is both phenomenologically rich and methodologically robust.

In addition, it is pertinent to note that, before data analysis, two parents and two caregivers with lived experience who were part of the project steering committee were trained by the lead researcher on the IPA process. The parents engaged in the coding exercise, including the review of emerging themes, and helped prioritize messages, including the selection of the media campaign’s name. The parents also commented on the proposed campaign materials and validated the final recommendations and jingle. All participants’ identifiable information, including real names, was removed and anonymized. Below is an example of a table summarizing themes and representative quotes (Table 1).

**Table 1:** Summary themes and representative quotes.

Transcripts	Emergent themes	Clustered superordinate themes
“OK, I think for me personally, I think transparency really matters. Like when information about side effects or the risk is not communicated properly, I may assume that something is being hidden. So, I think one thing that that should be done is transparency. They should be transparency as in you should explain what are the effects or the benefits of you giving your child that vaccine. I think if there is transparency it will go a long way.” (Bietorbi) “I think the introduction should just be like maybe a caption saying maybe the importance of vaccine or maybe vaccine prevents disease or maybe save life or then the body can be to address one of these issues, some of these concerns that we have raised. So just say maybe the just address maybe the mental or the concerns and the conclusion should just be like a call to action, maybe what exactly we need to do or what exactly, where exactly we need to go to get the right vaccine.” (Omolaye)	-Clarity -Transparency -Benefit of vaccines -Vaccine concerns -Informative content	Clarity, reassurance, and empowerment in vaccine communication
“OK, I think a 2-min video will be OK. If it’s like a radio Jingle or a short video under that 2 min with simple graphics that can be shared regularly.” (Bietorbi) “Okay, I think it should be in a few minutes. If it is a radio jingle in a vernacular. Then it should be spread morning, afternoon, evening. So, if it’s like 2 min in vernacular morning, afternoon, evening.” (Oghomena)	-Brevity -Conciseness -Consistency	Concise, culturally relevant, and easily digestible vaccine messaging
“Most of the mothers now are now online and they use TikTok and Instagram, mostly TikTok and Instagram. And if they could be radio jingles that can help.” (Teyhenah) “Yes, I will say news line, like network news, like this on Sundays, especially NTA News, they used to show all these stories there, and most people used to watch that. So, I think they can also use that media, like channel news, other news station and then let people know about this.” (Ivy)	-Social media platforms -Inclusive platforms	Navigating diverse and unequal media landscapes to aid uptake

## Findings

Based on the analyzed data underpinned by IPA, the findings revealed the following main themes: (i) Hostility, misinformation, and the breakdown of trust in vaccines; (ii) Faith, tradition, and the lived logic of alternative protection; (iii) Loss of confidence in professional competence and healthcare; (iv) Clarity, reassurance, and empowerment in vaccine communication; (v) Blending care and authority: gendered voices in building trust in vaccine messaging; and (vi) Navigating diverse and unequal media landscapes to aid uptake. The findings from the analyzed themes informed the final co-produced 2.45-min jingle message contained in Appendix Table 1. The analyzed themes are reported below.

### Hostility, misinformation, and the breakdown of trust in vaccines

A recurrent pattern in the participant’s lived experience is the perceived intersection of misinformation and hostility, which fuels the breakdown of trust in vaccines and thereby exacerbates hesitancy. This theme, therefore, captures how participants’ trust in vaccination becomes deeply unsettled when interactions with healthcare providers are hostile, dismissive, or unsafe. Across their accounts, fears fueled by frightening narratives merge with personal experiences of being talked over or reprimanded, creating a sense that the vaccination environment is not only unwelcoming but also potentially harmful. This leads participants to withdraw from services, avoid them, and question the integrity of the entire vaccination system. Talking about the former, relating to misinformation, one participant said:

There is conspiracy. There is a news that this COVID-19 vaccine that they brought then. It’s still in terms of reduced population and all that. […] and there’s an issue that happened in one of the states I read the news online. That a group of persons just pretend as nurses and they injected some children in the name of vaccination and the children, some of them gave up. You understand, their body system could not take it so. It’s still really cause issues. So, this vaccination stuff now is making me have two minds about vaccination now because some of them are using this as a channel of wicked. (Omotekoro).

The participant’s extract reveals a lived experience marked by deep uncertainty, fear, and moral tension as the participant navigates conflicting narratives about vaccination, with online stories of impersonated nurses harming children profoundly destabilizing perceptions of safety and trust. The participant’s reference to *“conspiracy”* and population-reduction narratives suggests that conspiracy frameworks serve as a dominant lens through which the participant makes sense of health interventions, providing emotional coherence amid confusion. At the same time, the vivid imagery of children *“giving up”* imbues the participant’s narrative with collective trauma and moral shock, transforming vaccination from a routine public health practice into a potential site of intentional harm. This appears to lead to an internal conflict—described as having *“two minds”*—as the participant grapples with the tension between social expectations to vaccinate and the destabilizing fear that vaccination may be used as a “channel of wicked,” signaling a profound breach of institutional trust. On a similar note, one participant said:

The medical practitioners, most of them, they are always angry. […] this nurse was about to give my child vaccine, and she was talking, she has been talking. So, when it got to my turn, and I asked her, what is this one that you want to give to my child? She got angry like, does she not know what she was doing or what? But I need to know because you have been talking, I feel you have been distracted. […] But she was angry. And now after giving my child that vaccine, my child got ill. So that kind of thing, […] already, I am scared now I am scared. I do not even want to go there. I do not even want to vaccinate my children anymore because I already have no trust for them. Asking them a simple question turns them off. So that is one of the issues. (Teyhenah).

The participant’s account conveys a deeply embodied experience of fear, mistrust, and emotional exhaustion emerging from repeated encounters with what is perceived as hostile or dismissive healthcare behavior. The participant narrative centers on a moment in which a nurse’s irritation, triggered simply by being asked to clarify the vaccine she was about to administer, becomes symbolic of a perceived wider pattern of medical practitioners being *“always angry,”* transforming a routine vaccination encounter into an emotionally unsafe space. The participant’s protective instincts as a caregiver intensify vigilance, particularly because the nurse’s distracted behavior is perceived as a threat to the child’s wellbeing, a fear seemingly validated when the child subsequently became ill. This event becomes a turning point, generating enduring avoidance and, progressively, mistrust across the entire health center, as mistrust appears to spread from the individual nurse to the healthcare system more broadly. The participant’s repeated use of words like *“scared”* and “*don’t even want to go there*” illustrates how a single negative interaction can escalate into a sustained withdrawal from vaccination services, shaped by a sense of being dismissed, disrespected, and emotionally violated when attempting to advocate for a child. Ultimately, this experience undermines the participant’s confidence in the safety and integrity of the vaccination process and contributes to a growing reluctance to vaccinate children.

### Faith, tradition, and the lived logic of alternative protection

A notable pattern in participants’ lived experiences was how they drew on deeply rooted religious beliefs and cultural healing traditions to make sense of childhood health, often positioning divine protection and ancestral remedies as trusted forms of care that reduce the perceived need for vaccination. Their accounts suggest that prayer, spiritual reliance, and long-standing herbal practices are significant factors through which protection, strength, and healing are understood and experienced. As a result, vaccination becomes entangled with questions of faith, identity, and cultural continuity, in which medical interventions are construed as unnecessary, misaligned, or even contradictory to the protective systems that have shaped families across generations. Talking about this, one participant said:

For me, I believe some of us, we so much believe. in divine protection, actually. So, taking vaccines sometimes negates that our faith. […] because we believe, when the child is sick, we will pray for the child to be healed and because that is, part of our religion in this part in Nigeria, it’s very, very true because we believe in God, we hold on to God, and we believe in that supernatural healing. And that’s one of the rules I would say have brought hesitation to taking vaccine. (Omolaye).

The participant’s account reveals how deeply spiritual beliefs shape the lived experience of childhood vaccination, highlighting an internal tension between faith-based understandings of protection and medical narratives of disease prevention. The emphasis on “divine protection” and “supernatural healing” reflects a meaning-making framework in which God is perceived as the primary source of safety, rendering vaccines at times unnecessary or even contradictory to expressions of faith. This creates a profound existential conflict: taking a vaccine may feel like a weakening of trust in divine intervention, particularly when prayer is seen as the rightful response to childhood illness. By situating this within Nigeria’s cultural and religious landscape, the participant appears to frame hesitancy as spiritually coherent—a response arising from long-standing traditions of relying on God in moments of vulnerability. Ultimately, in making sense of the participant’s experience, the narrative shows how vaccination decisions are intertwined with identity, belief, and communal religious practice, illustrating that hesitancy should be understood within the lived moral and spiritual world that provides meaning to those choices. Commenting on the role of culture, another participant said:

Culturally, we have herbs we can give to newborns to make them strong, so I rely on them a lot. I do not need medicine; I just need to pluck some leaves, get a few maggots, and put them in the water to bathe my baby. The leaves and mango tree: if you just cut it, wash it, heat it up, and give it to her to drink, she will be strong. That is what they used to treat us growing up, and it cures things like fever or malaria. So, no need for a vaccine. (Bedemi).

The participant’s extract highlights how culturally embedded healing practices form a powerful experiential foundation for understanding child health and lessening the perceived need for vaccination. The detailed description of using herbs, leaves, mango tree preparations, and traditional bathing rituals reflects not only alternative remedies but also a deeply embodied healthcare tradition passed down through generations, creating a sense of familiarity, trust, and continuity that biomedical interventions appear unable to replace easily. In making sense of the participants’ lived experiences, these practices are experienced as effective and sufficient—“*that is what they used to treat us growing up*”—and this lived history becomes a compelling narrative of resilience that positions cultural remedies as proven forms of protection against illnesses like fever or malaria. In this context, vaccines appear reinterpreted as unnecessary, even redundant, when measured against long-standing cultural experiences of healing that have sustained families for decades.

### Loss of confidence in professional competence and healthcare

Confidence in healthcare providers is crucial to ensuring optimal vaccine uptake. In this theme, participants’ trust in vaccination collapses when encounters with healthcare workers are experienced as careless, unskilled, or unsafe. Across their narratives, personal experiences of pain, improper procedures, and visible incompetence become powerful touchpoints that transform routine medical care into a source of fear and imagined danger. As these experiences accumulate, participants begin to doubt not only individual practitioners but also the entire system, leading to hesitancy, avoidance, and a deep-seated belief that medical experts can no longer be relied upon to protect their children. Commenting on this, one participant said:

I had an experience with a particular nurse when I gave birth to my baby. I went there to check the baby’s tongue and because she was eh very busy in doing so many things at the same time, she did what she was supposed to do properly, she did it very wrongly and that gave me one kind of mindset negatively against her and also against every one of them and we had so many different other issues with the particular nurse and […] because she is also involved with people that goes about sensitizing people concerning immunization here and there. And whenever I see her, I get this hesitation, I do not even care about the vaccine, I do not even care about the immunization because such a person cannot do something, do this, or do anything properly. (Erhuvwu).

The participant’s narrative reveals how a single negative encounter with a nurse becomes a catalyst for a much broader collapse of trust in healthcare and immunization systems. The experience of the nurse being rushed, distracted, and performing a procedure “very wrongly” is not only remembered as an isolated mistake but also internalized as evidence of systemic incompetence, generating a “*mindset negatively against her and also against every one of them*.” This moment of perceived carelessness—occurring at a vulnerable time shortly after childbirth—creates a lasting emotional imprint that is continually reinforced through further “issues” with the same practitioner, who paradoxically also serves as a public advocate for immunization. The participant experiences profound dissonance when a person believed to be incapable of providing competent care simultaneously occupies a role centered on promoting vaccine safety, leading to hesitation, avoidance, and a diminishing sense of obligation toward immunization. Another participant also recounts an experience and notes:

I think even the people in the hospitals giving vaccines, I somehow have a, let me say, terrible experience, terrible experience. […]I will not allow my child to just be injected anyhow in the hospital, even normal injection. Because even as an adult, I took an injection, and it almost paralyzed my legs. And it’s so crazy because I could not walk properly. And just exposing a small child to that kind of kind of thing. It’s very, very risky. I just imagine with my child that was given that injection, okay, in the name of vaccine, and that can paralyze the child. That is because the nurse administering the injection, probably she wasn’t professional enough or something happened. But I know it’s really, really affected me. So, I think the experts are no longer there or maybe the people there are not doing it with their mind or something. (Omolaye).

This account reflects an intensifying sense of vulnerability and distrust rooted in both personal and vicarious experiences of bodily harm associated with injections, which profoundly shape the participant’s interpretation of vaccination practices. Drawing on the lived experience of the participant, the memory of an injection that *“almost paralysed*” the participant’s legs becomes a powerful embodied reference point that amplifies fears about the risks posed to young children, transforming routine medical procedures into potential threats. This experience seems to undermine the perceived competence of healthcare workers, as the participant questions whether “*the experts are no longer there*” or whether staff are performing their duties without genuine care or professionalism. The idea that a nurse’s lack of expertise or attentiveness could lead to paralysis for a child exemplifies how isolated negative encounters can escalate into a generalized belief that the system itself is unsafe. Consequently, it appears that even standard injections are reinterpreted through a heightened sense of danger, prompting a protective withdrawal from vaccination services and reinforcing a broader narrative of declining trust in medical practitioners and the institutions they represent.

### Clarity, reassurance, and empowerment in vaccine communication

The design of media campaign interventions to address vaccine hesitancy requires strategic communication to foster effective change. In this theme, participants express a strong desire for vaccine information that is transparent, reassuring, and directly responsive to the fears circulating within their communities. Across their accounts, parents seek not only emotional validation but also practical knowledge—clear explanations of safety, purpose, and the right questions to ask—so they can feel confident and equipped in their decision-making. For these participants, trust in vaccination is rebuilt through communication that empowers rather than instructs, offering both reassurance and agency in navigating their children’s health. Talking about this, one participant said:

I think when it comes to the introduction, first of all, they need to acknowledge parents’ concerns without judging them. And also, they should emphasize that every parent wants the best for their child. […] Another point I would like to stress out is that they should briefly explain the purpose. That’s like they should briefly explain the purpose why they are carrying out the vaccine, like make them understand it’s to protect their children from preventable disease and it should also sound empathic and not authoritative. (Bietorbi).

This account reveals a profound yearning for communication that feels respectful, validating, and emotionally attuned, reflecting how deeply parental trust in vaccination hinges on interpersonal dynamics with healthcare professionals. The emphasis on acknowledging parents’ concerns “without judging them” speaks to an underlying experience of vulnerability where vaccination encounters are not merely informational but emotionally charged moments in which parents seek reassurance that their worries are legitimate and that their intentions are not misunderstood. The participant constructs a vision of good practice rooted in relational empathy—healthcare workers recognizing that “every parent wants the best for their child”—suggesting that mistrust often emerges not from the vaccines themselves but from encounters that feel dismissive or authoritarian. In making sense of the participant experience, a tension emerges between wanting to protect a child and needing professionals to create a communicative environment in which such protection feels shared rather than contested, revealing how emotional safety becomes a prerequisite for vaccine acceptance.

Commenting on the content of the media campaign intervention, another participant also said:

I will say how safe it is. The content should also contain how safe it is so that people will understand because most people do not want to take this because of the, they are not sure of how safe it is and then the importance. (Ivy).

This account highlights a deep need for reassurance about vaccine safety, revealing that uncertainty about what vaccines contain and how safe they truly are forms a central barrier to acceptance. The emphasis on including information about “*how safe it is”* reflects an underlying lived experience of anxiety and hesitation, shaped by a broader social context in which fear, rumors, and uncertainty circulate widely. For this participant, clarity about safety and purpose is not merely informational but emotionally stabilizing, offering a sense of grounding in situations where parents feel responsible yet unsure. The statement that “most people don’t want to take this because … they are not sure of how safe it is” captures collective apprehension, showing how individual fears are intertwined with wider community doubts. Another participant talking about the content of the media campaign intervention also said:

The topic can be vaccine does not kill, and then the person will be interested, by saying vaccine does not kill, they want to know why somebody is saying vaccine does not kill. And then in the body, you can talk about the kinds of vaccines that are supposed to be giving our children and the importance of it, like vaccinate your children and enjoy your children and all that. And then the conclusion can be where you get the vaccine, the kind of questions to ask when you go to a health center, you want to give the children vaccines. What are the questions to ask to be sure you are getting the right vaccine? (Teyhenah).

This account reveals a desire for vaccine communication that captures attention through reassurance while simultaneously addressing the underlying anxieties that shape parental hesitancy. The suggestion of a message like “vaccine doesn’t kill” reflects an acute awareness of the emotional landscape parents inhabit—one marked by fear, doubt, and exposure to alarming narratives—and illustrates how parents seek language that directly counters these unspoken worries. At the same time, the participant imagines an educational structure that moves beyond reassurance to provide concrete, practical information about different vaccines, their importance, and the questions parents should ask to ensure safe administration, indicating a deeper need for empowerment rather than the passive receipt of instructions. Underpinning this is a lived experience of wanting transparency and agency in vaccination encounters: participants not only seek to be convinced but also to be equipped, suggesting that trust is rebuilt not merely through messages of safety but through enabling parents to feel knowledgeable, vigilant, and involved in safeguarding their children’s wellbeing.

### Concise, culturally relevant, and easily digestible vaccine messaging

Brevity in media campaign messages is crucial to ensure optimal engagement. In this theme, participants expressed a desire for vaccine communication that is brief, engaging, and sensitive to the realities of limited attention and busy daily routines. Commenting on the need for conciseness, one male participant said:

So, my concern to that will be the lengths for the media company. I think any media that is above 2 min, 3 min is going to be boring because everybody just want to watch and get the key message from it. (Folarin).

In the preceding extract, the participant highlights a practical yet emotionally grounded concern about attention and engagement, revealing how parents interpret public health communication through the lens of everyday cognitive and time constraints. The emphasis on keeping media “*below 2 or 3 minutes*” reflects an underlying lived experience of information fatigue, where long or dense messaging feels overwhelming and risks disengagement. This preference for brevity is not simply about convenience; it represents a desire for clarity, efficiency, and immediate access to key messages in a landscape where parents already feel burdened, anxious, or uncertain about vaccination. In the participant’s meaning-making, effective communication appears to require respect for the realities of modern information consumption—capturing attention quickly, delivering reassurance efficiently, and avoiding formats perceived as tedious or dismissive of people’s limited mental bandwidth. Ultimately, this account underscores how the form of communication, not just the content, shapes trust, receptivity, and the willingness to engage with vaccine information. Another participant also notes:

I think a 2-min video will be OK. If it’s like a radio Jingle or a short video under that 2 min with simple graphics that can be shared regularly (Bietorbi).

On a similar vein, one participant said:

Okay, I think it should be in a few minutes. If it is a radio jingle in a vernacular.

Then it should be spread morning, afternoon, evening. So, if it’s like 2 min in vernacular morning, afternoon, evening. (Oghomena).

The lived experiences of participants reveal a desire for vaccine communication that is concise, accessible, and delivered in formats that fit the fast-paced rhythm of daily life, highlighting how attention and comprehension are deeply tied to the emotional climate surrounding vaccination. Their preference for short videos, jingles, and simple graphics—ideally under 2 min—appears to reflect an underlying experience of cognitive overload, where lengthy or complex messaging risks disengagement and further distances parents from vaccine information. By emphasizing the value of regular repetition across familiar platforms, particularly in vernacular language, participants express a need for communication that feels culturally grounded and easy to absorb, suggesting that trust is strengthened when messages resonate with people’s linguistic and social contexts.

### Blending care and authority: gendered voices in building trust in vaccine messaging

A dominant narrative emerging from the participants’ lived experiences concerns how they make sense of vaccine communication through the symbolic and emotional meanings they associate with female and male voices. For them, a female voice offers a nurturing, caregiving tone that resonates with mothers’ everyday involvement in child health. In contrast, a male voice brings a sense of shared responsibility and decision-making authority, particularly in households where fathers hold significant influence. Together, these voices create a communicative balance that feels emotionally authentic and socially grounded, helping to normalize vaccination within the family unit.

To me, a female voice and a male voice will be OK. The two voices is OK because most times females are more concerned about this vaccination. You understand? Because they are the mothers and they are the one taking care of the children. Just for the males to be there with them, to just supervise them and know what they are doing also. So, the two voices are needed. (Omotekoro).

The preceding extract indicates that the participant’s account reveals how voice selection in vaccine messaging carries symbolic weight, reflecting deeper beliefs about gendered caregiving roles and the relational dynamics that shape parental decision-making. Their emphasis on including both a female and a male voice suggests an experiential understanding that mothers often shoulder the emotional and practical responsibilities of childcare, making a female voice feel relatable and trustworthy, while a male voice represents supportive oversight and shared responsibility within the family. This dual-voice preference conveys a longing for balance—communication that validates the mother’s central role while also encouraging fathers to be present and engaged—ultimately reflecting a broader desire for vaccine messaging that mirrors the social realities of parenting. In this meaning-making process, the voices in a campaign become more than auditory choices; they are experienced as extensions of social identity, responsibility, and relational support, shaping how safe, relevant, and inclusive the message feels to parents. Another participant also notes:

Personally, I feel for childhood vaccination, I would prefer a female voice, especially as a motherly or caring one, you understand? So, the reason is that in many households, mothers are more directly involved in child healthcare decisions and clinic visits. And however, I also think that including a male voice is also important, especially in communities where fathers influence is key and they are the major family, they are the one that gives the major family decisions. So, if fathers see other men supporting vaccination, it can normalize the decision and also reduce resistance. So ideally for me, a female voice is for emotional connection and caregiving tone, while a male voice for shared parental responsibility and broader household influence. (Ibifagha).

The participant’s account reveals a deeply considered understanding of how voice, tone, and gender representation in vaccine messaging intersect with lived family dynamics and cultural expectations around caregiving. Their preference for a female voice—associated with “a motherly or caring” quality—reflects a phenomenological recognition that mothers often carry the emotional labor and physical responsibility of child healthcare, making a female voice feel naturally attuned to parents’ anxieties and relational needs. At the same time, the participant’s insistence on also including a male voice highlights how communication is experienced within broader social structures, particularly in contexts where fathers hold significant decision-making authority; hearing other men endorse vaccination can, in their view, help normalize acceptance and reduce resistance. This dual-voice model appears to serve as a symbolic representation of shared parental responsibility, expressing a desire for messaging that mirrors the realities of household influence and family power dynamics. In making sense of the participant’s perspective, the choice of voice in a campaign is not a superficial design decision but a deeply relational one—capable of engaging emotional connection, affirming caregiving roles, and shaping whether vaccine messages resonate within the complex social fabric of family life.

### Navigating diverse and unequal media landscapes to aid uptake

Ensuring targeted and widespread dissemination that reaches the affected population is crucial to changing behavior relating to childhood vaccine hesitancy. This theme captures how participants make sense of vaccine communication in light of the practical realities of mothers’ media access, revealing a landscape shaped by both digital engagement and persistent infrastructural inequities. Their accounts show that while many mothers now use platforms like TikTok and Instagram, others—particularly in rural communities—still rely heavily on radio and community-based channels, making a single communication strategy insufficient. Thus, the findings reflect a lived recognition that effective messaging must be flexible, culturally attuned, and delivered across multiple accessible platforms to truly reach and support all mothers, regardless of location or resources.

Radio can also be used also because we have the people that stay in the rural areas. They mostly use radios. Some of them does not have the Android phone to WhatsApp and this media stuff. Some normally listen to radios like me that I actually go to the rural areas and most times I spend time there and they normally put their radios up there to listen to music, news and listening to many things. So, it’s few of them that might use Android and all that. […] So, you can also use radio, pastors and schools also ahead. You know, even in the rural area there are schools there, so the children can get this vaccines. (Oghomena).

The participant’s extract reveals how decisions about communication channels are deeply shaped by lived familiarity with rural contexts, where access to technology, media habits, and community structures differ significantly from urban settings. The emphasis on radio emerges from an embodied understanding of everyday rural life—where people “mostly use radios,” lack smartphones, and gather information through communal listening spaces—highlighting a desire for vaccine messaging that aligns with the realities of people’s environments rather than abstract assumptions. By drawing on personal experience of spending time in rural areas, the participant positions radio, pastors, and schools as trusted and accessible pathways through which messages can flow naturally within existing rhythms of daily life, suggesting that effective communication must work through the social institutions people already rely on. In making sense of the participants’ lived experiences, an effective media campaign appears to require the use of culturally embedded sources of authority and communication infrastructures that genuinely reach them. Another participant also said:

For me, I will still make a reference to the northern part of Nigeria. The perfect, what’s it called, social media that should be used should be the radio jingle in the sense that […] mothers are the ones taking care of the children, so if most of our mothers… do not have access to social media […], radio will be of good advantage to pass those, to disseminate this information to them. (Ighojiroro).

The account reveals a deeply contextual understanding of how communication must align with the lived realities of mothers in northern Nigeria, where limited access to smartphones and social media shapes the flow of health information. The preference for radio jingles reflects an experiential awareness that radio remains a trusted and accessible medium in many households, especially for mothers who bear primary responsibility for children’s health yet may lack the digital tools that urban populations rely on. By highlighting that radio offers “*good advantage”* for disseminating information, the participant positions vaccine messaging as being embedded in cultural, infrastructural, and gendered patterns of daily life. Thus, the account underscores a belief that effective communication must meet mothers where they are—through familiar channels, in accessible formats, and within the rhythms of their everyday routines—suggesting that trust in vaccination is strengthened when messages travel through media that genuinely reach and resonate with those most involved in child health decisions. Another participant, while reflecting on other social media platforms, also said:

Most of the mothers now are now online and they use TikTok and Instagram, mostly TikTok and Instagram. And if they could be radio jingles that can help (Lavina).

This account reflects an evolving awareness of how mothers are increasingly navigating both digital and traditional communication spaces, revealing the layered media environment through which vaccine information must travel. The observation that “*most of the mothers now are online*” and actively use TikTok and Instagram highlights a shift in everyday practices, where social media has become a central space for connection, entertainment, and health-related exposure. However, the simultaneous endorsement of radio jingles suggests a meaning-making process that recognizes the heterogeneity of mothers’ circumstances—acknowledging that while some are digitally engaged, others still rely on radio as a familiar, trusted, and accessible channel. This dual emphasis illustrates a lived understanding that no single medium is sufficient; rather, effective vaccine communication must mirror the diverse realities of mothers who oscillate between online platforms and traditional broadcast media depending on accessibility, routine, and social context. Ultimately, the participant’s account appears to convey the belief that trust and reach are strengthened when messaging aligns with mothers’ actual media habits, combining modern digital platforms with inclusive, widely accessible channels like radio.

## Discussion

The purpose of this study was to examine how parents or caregivers of children who are not or only partially up to date with immunizations can inform the development and co-production of a media campaign intervention that effectively promotes childhood vaccine uptake and reduces hesitancy. Informed by an IPA, this study reported several superordinate themes, with findings discussed in relation to the literature.

First, the findings of this study illustrate the multidimensional and deeply situated nature of vaccine hesitancy among caregivers, echoing the broader patterns observed in Nigerian and sub-Saharan African contexts. We found that issues around hostility, misinformation, and the breakdown of trust in vaccines exacerbate hesitancy. Participants’ accounts reveal how mistrust in healthcare workers, negative interpersonal encounters, and perceived breaches in professional competence substantially undermine confidence in vaccination. This aligns with previous studies in the Nigerian context, which show that poor provider attitudes, hurried consultations, and inadequate communication are major drivers of parental resistance to immunization services (24). As our study findings show, descriptions of nurses as dismissive, distracted, or careless intersect with mothers’ experiences and serve as powerful emotional anchors, shaping perceived mistrust and supporting research showing that interpersonal disrespect and poor communication erode vaccination acceptance more strongly than medical concerns themselves (25).

Second, the study findings highlight how religious beliefs and cultural healing traditions profoundly shape caregivers’ meaning-making around child health. Participants articulated how faith in divine protection and reliance on supernatural healing can make biomedical vaccination appear contradictory or unnecessary. This aligns with previous research showing that religious convictions, belief in divine will, and spiritual interpretations of illness remain strong determinants of vaccine hesitancy in Nigeria (26), particularly when caregivers view vaccines as competing with, rather than complementing, divine authority. Similarly, participants’ reliance on herbal remedies, traditional bathing practices, and other generationally transmitted healing methods reflects a broader cultural framework in which indigenous health practices are viewed as effective, trusted, and identity-affirming. This corresponds with previous literature findings revealing low perceived susceptibility to vaccine-preventable diseases and strong confidence in traditional remedies among rural Nigerian caregivers, which contribute to reduced uptake of biomedical immunization (27, 28).

A third significant theme emerging from the narratives concerns the centrality of communication—its tone, clarity, format, cultural relevance, and delivery channel. This study found a consistent emphasis on the need for empathetic, non-authoritarian messaging that acknowledges parental fears and treats parents as partners rather than passive recipients of care. This resonates with previous literature showing that culturally appropriate communication strategies, community engagement, and improved provider–patient relational skills are essential for addressing hesitancy in Nigeria (28). In particular, parents’ desire for simple, clear explanations about vaccine safety aligns with findings from culturally tailored interventions, which show that accessible, localized messaging strengthens trust and improves knowledge, even if it does not immediately translate into increased vaccine uptake. The preference for short videos, jingles, and vernacular-language content also aligns with evidence that brief, culturally embedded audio formats—especially those integrating local music or voices—enhance message relatability and emotional resonance for rural women and mothers of young children (28, 29).

Our study also found that communication preferences were shaped by uneven access to information technologies. While some participants emphasized platforms such as TikTok and Instagram for younger, digitally connected mothers, others foregrounded radio as a critical medium for rural communities lacking smartphones or internet access. This variation mirrors current public health challenges: radio remains the most widely accessible medium across many Nigerian regions, especially among rural women, making it a key vehicle for disseminating maternal and infant health information. The literature equally underscores that successful vaccine campaigns should diversify their communication channels, ensuring that both digital and traditional media are strategically used to reach heterogeneous audiences (28).

Finally, participants’ reflections point to broader issues of trust—trust in medical institutions, in government health systems, and in the professionals responsible for administering vaccines. This distrust appears to be shaped by historically rooted experiences, including past controversies, misinformation, and systemic inadequacies that continue to inform contemporary hesitancy. Recent analyses of Nigerian vaccine hesitancy similarly note the enduring influence of historical medical scandals, misinformation, and socio-cultural resistance on public distrust, particularly in northern regions with long-standing suspicion of government-led health interventions (34). Collectively, the findings of this study reinforce the understanding that vaccine hesitancy appears embedded in lived histories, relational encounters, socio-cultural identities, and the practical realities of everyday life.

### Strengths and limitations of this study

This study acknowledges some limitations, including reliance on a small, self-selecting sample, which limits the transferability of the findings beyond the specific communities represented. As the data are based entirely on self-reported experiences, accounts may be shaped by recall bias or personal interpretation, which is common in qualitative research. A key limitation of this study is that participants were recruited from a single state in Nigeria and predominantly comprised individuals with higher levels of education. This restricts the diversity of perspectives captured, as women with little or no formal education—who may face different socio-cultural, informational, and access-related barriers—are underrepresented. Consequently, the findings may not fully reflect the broader population of women, particularly those in underserved or rural settings, where different factors may shape reluctance to vaccinate children. Future research should include a more diverse, representative sample by recruiting participants across multiple regions and varying educational backgrounds. Particular emphasis should be placed on engaging women with limited or no formal education to capture better the full spectrum of beliefs and barriers influencing childhood vaccine uptake. This will enhance the transferability of findings and support the development of more inclusive, context-sensitive public health interventions.

Notwithstanding the limitations, the strength of this study lies in its use of IPA, which provides an original and highly significant lens for exploring how parents and caregivers make sense of childhood vaccination and their role in co-creating a media intervention. IPA foregrounds participants’ *lived experience* and their personal meaning-making, enabling this study to capture the emotional, cultural, and relational complexities that shape hesitancy—dimensions often overlooked in survey-driven approaches. By enabling a deep exploration of mistrust, communication needs, religious beliefs, and culturally rooted health practices, IPA offers a uniquely rich foundation for designing a media campaign that resonates authentically with parents’ and caregivers’ realities, ensuring that policy and communication strategies are grounded in the voices and meanings of those they aim to influence.

### Implications for policy

The findings of our study point to several notable distinctions between vaccine hesitancy and access and service-quality barriers. For example, as the discussion shows, some findings relate to mistrust, misinformation, faith, and traditional remedies, while others point to unequal media access, rural communication infrastructure, and negative healthcare encounters, with policy implications. In essence, our study has important policy implications for policymakers, as it highlights the need to address confidence, communication, convenience, and service experience. This can be achieved by prioritizing empathy-based communication, trust-building, and culturally grounded engagement to address vaccine hesitancy in Nigeria, as national evidence indicates that mistrust, poor provider communication, and cultural–religious beliefs remain major barriers to uptake.

Policymakers are also encouraged to invest in multi-channel communication strategies, as the study identifies the need for short, vernacular, repeated, emotionally reassuring, and culturally credible messages delivered through both digital platforms and radio. This is to ensure both rural and digitally active mothers receive accessible, relatable information that can instill trust, reduce childhood vaccine hesitancy, and promote uptake. As such, this could be translated into a concise campaign logic as follows: trusted messenger, core message, target audience, platform mix, frequency, language adaptation, and evaluation outcome to enable policymakers’ practical implementation. In addition, this co-produces a jingle to encourage childhood vaccine uptake, which is intended to be tested in a randomized controlled trial to determine its feasibility, acceptability, effectiveness, and cost-effectiveness, as appropriate, compared with existing usual public health interventions.

Furthermore, policymakers are encouraged to address service-quality and structural access barriers that limit vaccine uptake. This also includes poor healthcare provider behaviors, which further exacerbate vaccine hesitancy. Strengthening healthcare worker training on respectful, clear, and non-authoritarian interactions is essential, as supportive provider behavior may increase parental confidence and vaccine acceptance.

## Conclusion

In conclusion, this study advances understanding of media campaign interventions to encourage childhood vaccine uptake. The findings demonstrate that childhood vaccine hesitancy is a deeply layered phenomenon shaped by concerns about medical safety, as well as by parents’ lived experiences, interpersonal encounters, cultural identity, and religious perspectives. Through IPA, the research uncovered how mistrust in healthcare workers, negative clinic interactions, communication barriers, and limited access to appropriate information intersect with faith and long-standing traditional healing practices to influence vaccination decisions. These findings highlight that effective interventions should move beyond generic public-health messaging and instead engage with parents’ emotional realities, cultural logics, and preferred communication channels, including radio, vernacular media, short videos, and trusted community voices. By foregrounding parents’ perspectives in the co-creation of a media campaign, this study provides a richer, more human understanding of vaccine hesitancy. It offers a foundation for designing communication strategies that are not only informative but also respectful, culturally resonant, and sensitive to the social context in which caregivers make decisions. Ultimately, the research underscores that rebuilding trust in vaccination requires relational, context-aware approaches that embed the beliefs, experiences, and everyday constraints of the families whom such interventions aim to serve, thereby encouraging childhood vaccination uptake and preventing hesitancy.

## Data Availability

The original contributions presented in the study are included in the article/supplementary material, further inquiries can be directed to the corresponding author.

## Appendix

**Table A1:** Co-produced jingle to encourage childhood vaccine uptake: Assured awareness campaign to address parental hesitancy and encourage childhood vaccine uptake (ASSURE).

Length	“PROTECT Their Tomorrow—Vaccinate Today” (PROTECT) jingle to encourage childhood vaccine uptake
2:45	Fellow parents and caregivers, today I come to you with an important message on child vaccination! Every child deserves a healthy start in life—but many dangerous diseases like measles, polio, whooping cough, and meningitis still threaten our children. These illnesses can cause lifelong disability and even death. The good news is that they are preventable. Why childhood vaccination matters: Vaccines save lives, and they are not designed to reduce the population. Children who miss routine immunization are at a higher risk of severe complications from common diseases. Diseases we think are gone can return. When fewer children are vaccinated, outbreaks happen—and spread quickly in our communities. Vaccinated children grow stronger. They attend school more regularly, learn better, and thrive into adulthood. Vaccination is free in Nigeria. Every parent can access it at government hospitals and primary health centers nationwide. Failing to vaccinate can lead to: Permanent paralysis from polio, blindness or brain damage from measles, pneumonia, seizures, and death (from whooping cough and meningitis). It can also lead to costly hospital bills and emotional stress—completely avoidable with vaccines. What action can you take? Your decision as a parent can protect not only your child but also your entire community. So, be a hero for your child. Visit the nearest hospital or health center today and complete your child’s immunization schedule. It’s safe. It’s free. It’s your right—and your child’s future. Together, let us build a healthier Nigeria. Protect Their Tomorrow —Vaccinate Today!

## References

[1] World Health Organization (2024) Global childhood immunization levels stalled in 2023, leaving many without life-saving protection. Available online at: https://www.who.int/news/item/15-07-2024-global-childhood-immunization-levels-stalled-in-2023-leaving-many-without-life-saving-protection (accessed March 20, 2026)

[2] Mohammed Y Reynolds HW Waziri H Attahiru A Olowo-Okere A Kamateeka M . Exploring the landscape of routine immunization in Nigeria: a scoping review of barriers and facilitators. Vaccine. (2024) 20:100563. doi: 10.1016/j.jvacx.2024.100563, 39430738 PMC11488437

[3] Sabo A Alzoubi MM Garba A Saidu AY Usman US Saulawa IM . Determinants of routine immunization coverage among under-five children in Jigawa state, Nigeria. BMC Public Health. (2025) 25:2065. doi: 10.1186/s12889-025-23363-2, 40461991 PMC12135541

[4] Eslamkhah S Aslan ES Yavas C Akcalı N Batur LK Abuaisha A . Mpox virus (MPXV): comprehensive analysis of pandemic risks, pathophysiology, treatments, and mRNA vaccine development. Naunyn Schmiedeberg's Arch Pharmacol. (2025) 398:6143–63. doi: 10.1007/s00210-024-03649-9, 39777535

[5] Halane S Ahmed A Ahmed MM Hersi MD Sani J. Assessing prevalence and regional disparities in zero-dose immunization among children aged 12–23 months in Somalia. J Epidemiol Global Health. (2025) 15:59. doi: 10.1007/s44197-025-00395-w, 40227511 PMC11996740

[6] Sayem ASM Musuka G Atuhebwe PL Dadari I Siddique AR. Childhood vaccination catch-up and recovery plans for mitigating immunity gap post the COVID-19 pandemic: a case study of selected African countries. Vaccine. (2025) 61:127328. doi: 10.1016/j.vaccine.2025.127328, 40450850

[7] Tafuri S Cuscianna E Bianchi FP. Prevalence of poliovirus neutralizing antibodies in Italian population: a systematic review and meta-analysis. Vaccine. (2023) 41:4057–63. doi: 10.1016/j.vaccine.2023.04.047, 37121798

[8] Bianchi FP Cuscianna E Di Lorenzo A Daleno A Mele F Marra M . A new paradigm of hospital care for SARS-COV-2 patients in the post-emergency phase in Italy. Annali Igiene Medicina Preventiva Comunità. (2023) 35:250–3. doi: 10.7416/ai.2022.2546, 36222605

[9] Adeyanju GC. Immunisation decision-making and barriers to vaccine uptake among children under-5 in limited-resource settings. Sci Rep. (2025) 15:44146. doi: 10.1038/s41598-025-33121-4, 41419591 PMC12717033

[10] Bolarinwa OA Salaudeen GA Lar L Alhaffar M Abdelmagid N McGowan CR . Barriers and enablers to childhood immunization in high zero-dose burden communities in Kano and Lagos states, Nigeria. Vaccine. (2025) 64:127754. doi: 10.1016/j.vaccine.2025.127754, 40946351

[11] Sani J Halane S Ahmed MM Ahmed AM Hersi MD. Regional disparities and maternal sociodemographic determinants of full immunization coverage among children aged 12–23 months in Nigeria: insights from NDHS 2018. Pediatr Health Med Ther. (2025) 16:157–70. doi: 10.2147/PHMT.S520721, 40655295 PMC12255271

[12] Nigeria Demographic and Health Survey (2024) Nigeria 2024 Demographic and Health Survey Summary Report. Available online at: https://dhsprogram.com/pubs/pdf/SR294/SR294.pdf (accessed March 20, 2026)

[13] Ahmad A. (2025). The mother of all campaigns": inside Nigeria’s 100 million child vaccine drive. Gavi: Vaccine Alliance. Available online at https://www.gavi.org/vaccineswork/mother-all-campaigns-inside-nigerias-100-million-child-integrated-vaccine-drive#:~:text=Although%20the%20country%20has%20made,meant%20for%20the%20same%20population (accessed March 20, 2026)

[14] Balogun FM Omobowale OC Akindolire AE. Enablers and barriers to older women's support of infant immunization timeliness and completion in selected urban slum communities of Ibadan, Nigeria. Vaccine. (2025) 25:100668. doi: 10.1016/j.jvacx.2025.100668

[15] Abreu L. Schrage P. Adeyanju G. C. Jalo R. I. Abulfathi A. A. Bello M. M. . (2025). Caregivers’ qualitative insights on trust, resilience and vaccine hesitancy shaping child health in conflict-affected Northeast Nigeria. [Epubh ahead of preprint]. doi: 10.1186/s13031-026-00753-w PMC13049947 41764511

[16] Oladosu MA Abah MA Omoseeye SD Musa Z Ezeamii PC Etus PC . Determinants and interventions for vaccine hesitancy in rural communities: a global narrative review of socio-cultural, institutional, and infrastructural barriers. Int J Adv Biol Biomed Res. (2026) 14:171–90. doi: 10.48309/ijabbr.2026.2065686.1634

[17] Aminu SB Musa M Kashim YA. Challenges in reaching full coverage of routine immunization in Nigeria: a review. Asian J Immunol. (2026) 9:11–22. doi: 10.9734/aji/2026/v9i1180

[18] Ike TJ Ezekiel Jidong D Nwanzu CL Ntaji MI Adamu DK Ayobi KO . Problem management plus mindfulness informed legal education (PM+ MiLE) intervention for addressing gender-based violence and improving parenting in south-South Nigeria: a randomised controlled trial. J Interpers Violence. (2025):08862605251393732. doi: 10.1177/08862605251393732, 41346078

[19] Ike TJ Jidong DE Ike ML Ayobi EE Ike PR Ayobi OK . Public attitudes and perceptions towards gender-based violence in Nigeria: a mixed method study. J Gender Based Violence. (2025):1–22. doi: 10.1332/23986808y2025d000000083

[20] Ike TJ Jidong DE Ayobi EE. Women’s perceptions of domestic, intimate partner violence and the government’s interventions in Nigeria: a qualitative study. Criminol Crim Just. (2023) 23:791–811. doi: 10.1177/17488958221128933, 37585072

[21] Bryman A. Social Research Methods. Oxford: Oxford University Press (2016).

[22] Burr V. Social Constructionism. London: Routledge (2024).

[23] Palmer M Larkin M De Visser R Fadden G. Developing an interpretative phenomenological approach to focus group data. Qual Res Psychol. (2010) 7:99–121. doi: 10.1080/14780880802513194

[24] Oku A Oyo-Ita A Glenton C Fretheim A Ames H Muloliwa A . Perceptions and experiences of childhood vaccination communication strategies among caregivers and health workers in Nigeria: a qualitative study. PLoS One. (2017) 12:e0186733. doi: 10.1371/journal.pone.0186733, 29117207 PMC5678719

[25] Jibrin AM Ugu AJ Babylon P Aliyu AS. A qualitative assessment of non-compliance to polio vaccination across diverse regions of Nigeria. Int J Med Sci Health Res. (2025) 9, 163–176. Available online at: https://www.researchgate.net/profile/Babylon-Philemon/publication/393428963_A_Qualitative_Assessment_of_Non-compliance_to_Polio_Vaccination_Across_Diverse_Regions_of_Nigeria/links/687349ebae516743559ca657/A-Qualitative-Assessment-of-Non-compliance-to-Polio-Vaccination-Across-Diverse-Regions-of-Nigeria.pdf

[26] Ohammah L. M. (2020) Sociopolitical Determinants of Parental Acceptance of Childhood Vaccination in Abuja, Nigeria (Doctoral Dissertation, Walden University)

[27] Anyabolu OI. Understanding Caregivers' Perceptions of Childhood Immunization. Minneapolis, Minnesota: Walden University (2016).

[28] Iwu CA Uwakwe K Ejikem ME Oluoha U Nwaigbo E. Cultural tailoring of vaccination messages: leveraging culturally adapted audio messaging in the promotion of maternal and infant vaccination uptake in rural communities in Nigeria. BMC Public Health. (2025) 25:4151. doi: 10.1186/s12889-025-25293-5, 41299334 PMC12659619

[29] Agbede GT Emezirinwune D Adedokun TL Idowu-Collins P. Vaccine hesitancy in Nigeria: overcoming cultural, linguistic and religious obstacles. Inf Impact J Inf Knowl Manag. (2024) 15:153–68. doi: 10.4314/iijikm.v15i1.12

[30] Smith J. A. Flowers P. Larkin M. (2009). Interpretative phenomenolog‐ ical analysis: Theory, method and research. London, UK: Sage.

[31] Eatough V. Smith J. A. (2017). Interpretative phenomenological analysis. The Sage handbook of qualitative research in psychology. London: SAGE. 2:193–209.

[32] Charmaz K. (2014). Constructing grounded theory. 2nd ed. London: SAGE.

[33] Lincoln Y. S. Guba E. G. (1985). Naturalistic inquiry. London: SAGE.

[34] Suru S. M. Ugwu C. E. (2025). Addressing malaria vaccine hesitancy in Nigeria: lessons from the Pfizer-Kano incident and COVID-19 vaccination. Front. Trop. Dis. 6, 1691239.

